# Optimized Production of Vanillin from Green Vanilla Pods by Enzyme-Assisted Extraction Combined with Pre-Freezing and Thawing

**DOI:** 10.3390/molecules19022181

**Published:** 2014-02-19

**Authors:** Yanjun Zhang, Limei Mo, Feng Chen, Minquan Lu, Wenjiang Dong, Qinghuang Wang, Fei Xu, Fenglin Gu

**Affiliations:** 1 Spice and Beverage Research Institute, Chinese Academy of Tropical Agricultural Sciences (CATAS), Wanning 571533, China; 2 National Center of Important Tropical Crops Engineering and Technology Research, Wanning 571533, China; 3 Key Laboratory of Genetic Resources Utilization of Spice and Beverage Crops, Ministry of Agriculture, Wanning 571533, China; 4 College of Food Science and Technology, Hainan University, Haikou 570100, China; 5 Department of Food, Nutrition and Packaging Sciences, Clemson University, Clemson, SC 29634, USA

**Keywords:** green vanilla, pectinase-assistant extraction, pre-freezing and thawing, response surface methodology, vanillin production

## Abstract

Production of vanillin from natural green vanilla pods was carried out by enzyme-assisted extraction combined with pre-freezing and thawing. In the first step the green vanilla pods were pre-frozen and then thawed to destroy cellular compartmentation. In the second step pectinase from *Aspergillus niger* was used to hydrolyze the pectin between the glucovanillin substrate and β-glucosidase. Four main variables, including enzyme amount, reaction temperature, time and pH, which were of significance for the vanillin content were studied and a central composite design (CCD) based on the results of a single-factor tests was used. Response surface methodology based on CCD was employed to optimize the combination of enzyme amount, reaction temperature, time, and pH for maximum vanillin production. This resulted in the optimal condition in regards of the enzyme amount, reaction temperature, time, and pH at 84.2 mg, 49.5 °C, 7.1 h, and 4.2, respectively. Under the optimal condition, the experimental yield of vanillin was 4.63% ± 0.11% (dwb), which was in good agreement with the value predicted by the model. Compared to the traditional curing process (1.98%) and viscozyme extract (2.36%), the optimized method for the vanillin production significantly increased the yield by 133.85% and 96%, respectively.

## 1. Introduction

Originated from Mexico, vanilla is one of the most important and popular aromatic spices, now widely planted in tropical and subtropical areas [1]. It has attracted much interest because of its multiple important applications in herbal cigarettes, alcoholic beverages, foodstuffs, cosmetics and aromatherapy [2,3]. Fully mature vanilla is called vanilla beans which are the fruits of *Vanilla planifolia* Andrews (Orchidaceae). The original unprocessed vanilla beans are flavorless until they are processed under a laborious curing process that lasts more than 6 months to give them their characteristic aroma [4,5].

The single most characteristic component of vanilla flavor is vanillin (4-hydroxy-3-methoxybenzaldehyde) [5,6]. Since vanilla is one of the most expensive and desirable spices, efforts to increasing the production of natural, effective, and safe vanillin have never stopped because the application of synthetic vanillin in valued food products is restricted due to the concerns about its safety [7,8]. Therefore, a market-driven strong need for high-quality natural vanillin has continuously pushed up its price in recent years.

Plant materials are rich in natural phytochemicals such as flavour compounds. They are difficult to separate because of their chemical sequestration, mainly by natural polymers such as pectins, which leads to incomplete solvent extraction [1,9]. Vanilla belongs to the monocots and is rich in pectin substances [10]. The glucovanillin content in green vanilla beans ranged from 10% to 15% (dry weight basis) which could produce between 4.8% and 7.3% vanillin [4]. The natural vanilla beans are flavorless because: (1) glucovanillin is exclusively located in the placentae and papillae while β-glucosidase is concentrated in the cytoplasm of mesocarp and endocarp, so the enzymatic reaction between the glucovanallin substrate and the enzyme cannot happen; (2) it is regulated by cellular compartmentation [11,12]. As a result, different kinds of processing technologies to increase the vanillin production from natural vanilla beans have been investigated. Partial cell destruction by mechanic forces and/or the use of enzymes to make intracellular compounds more readily available for separation and solvent extraction have been studied. The ‘scalding’ and ‘sweating’ stages of vanilla curing initiate the release of vanillin due to cellular decompartmentation as recently reported by Pérez-Silva *et al.* [6] The cellular compartmentation of the green vanilla beans could be disrupted by freezing and thawing so as to increase the extractability of vanillin as reported by Odoux *et al.* [12], and by Ansaldi *et al.* who filed a patent [13]. Some other investigators have studied the treatment of cured beans using exogenous pectinase and β-glycosidase to increase the vanillin yield [1,14]. Other researchers have investigated the effects of exogenous cellulase, pectinase, β-glycosidase and an enzyme extracted from tea leaf on the vanillin yield and vanilla flavor using green vanilla beans as raw material [2,3,5,9,15]. In addition, cellular disruption by mechanical means and freezing together with added pectinase, cellulase, and β-glusosidase increased the vanillin yield from the green vanilla beans as reported by Perera and Owen [4]. By contrast, the traditional extraction methods for vanillin are commonly associated with longer extraction times, higher temperatures and more organic solvent consumption, despite their lower extraction efficiency. In recent years, enzyme-assisted extraction has undoubtedly emerged as a new technology applied in many kinds of processing in the food industry since it has many advantages, such as faster extraction, higher recovery, reduced solvent usage, simplified processing and lower energy consumption when compared to the non-enzymatic methods. Thus, enzyme-assisted extraction has been considered an effective, feasible and practical technique for the extraction of vanillin. Immobilization shows many advantages for enzymes such as enhancing enzyme activity, stability, resistance to inhibition, selectivity towards non-natural substrates and so on [16,17]. Enzyme immobilization technology could be one way to further improve the process of enzyme reaction. However, to the best of our knowledge, none of the reported investigations involve the use of pectinase-assisted extraction combined with pre-freezing and thawing to produce vanillin from vanilla beans. Furthermore, compared with many other studies of the enzyme-assisted extraction in other applications, little effort has been devoted to optimizing the production of vanillin from green vanilla beans with this method.

Response surface methodology (RSM) is a powerful and efficient mathematical approach that enables evaluation of several process parameters such as time, temperature, enzyme type and concentration [18,19,20,21]. It can determine parameters’ interactions, the best optimal condition of the process, and the effect of factors on characteristic properties [19,21,22]. In addition, it is less laborious and can provide more information of the variables than other approaches. RSM has been found to be successful and economical during optimization of various industrial extraction processes [18,23].

The purpose of the present study was to produce vanillin from vanilla beans by using the technique of pectinase-assisted extraction combined with pre-freezing and thawing, and then optimize the process. Compared to previous research, enzyme-assisted extraction combined with pre-freezing and thawing could significantly increase the vanillin content from green vanilla pods. The green vanilla pods were first pre-frozen and then thawed in order to destroy cellular compartmentation. The extraction reactions referred to various aspects including enzyme amount, temperature, time, and pH. The pectinase amount, reaction temperature, time, and pH were identified as key factors influencing the extraction of vanillin based on our preliminary experiments. In the first part of this study, the process for extraction of vanillin from the vanilla beans was optimized by employing a central composite design (CCD) (four factors and five levels) and RSM to study the effects of the abovementioned variables on vanillin production. In the second part, the experimental model was validated by the optimized method. Finally, the method was compared with the traditional curing and viscozyme extraction methods.

## 2. Results and Discussion

As shown in Table 1, the concentrations of glucovanillin and vanillin in fresh vanilla beans (FVB) are 11.38% and 0.21%, respectively. This indicated that very little glucovanillin (<5%) was hydrolyzed before any processing. Even the green vanilla beans were processed after freezing and thawing (FT), the concentrations of glucovanillin and vanillin were 10.21% and 0.67% (Table 1), respectively. The comparison of vanillin liberation from green vanilla pods after enzymatic hydrolysis with and without freezing pretreatment has been done in the preliminary test. It was found that the vanillin content (1.22%) was significantly lower without freezing pretreatment than that (4.62%) of pectinase hydrolysis with freezing pretreatment. Therefore, the freezing pretreatment was needed in this work. Furthermore, it was emphasized that the enzyme reaction conditions needed to be further optimized by the response surface method based on the freezing pretreatment and thawing in this work.

**Table 1:** Quantification of major constituents in different samples determined by HPLC (dwb).

Treatments	*p-*Hydroxybenzoic acid (%)	*p*-Hydroxybenzaldehyde (%)	Vanillic acid (%)	Glucovanillin (%)	Vanillin (%)
FVB	-	-	-	11.38 ^a^	0.21 ^f^
FT	-	-	-	10.21 ^b^	0.67 ^e^
FTV	0.011 ^b^	0.024 ^c^	0.038 ^d^	8.58 ^c^	0.98 ^d^
PVE	0.021 ^a^	0.051 ^b^	0.078 ^b^	1.39 ^f^	4.62 ^a^
VVE	0.014 ^b^	0.026 ^c^	0.043 ^c^	4.63 ^e^	2.36 ^b^
SVE	0.023 ^a^	0.094 ^a^	0.15 ^a^	6.42 ^d^	1.98 ^c^

“-” Not detected. Values followed by the same letter in the same column are not significantly different (*p* < 0.05).

### 2.1. Effect of the Single Factor Test

#### 2.1.1. Effect of the Enzyme Amount on the Vanillin Content

**Figure 1:**
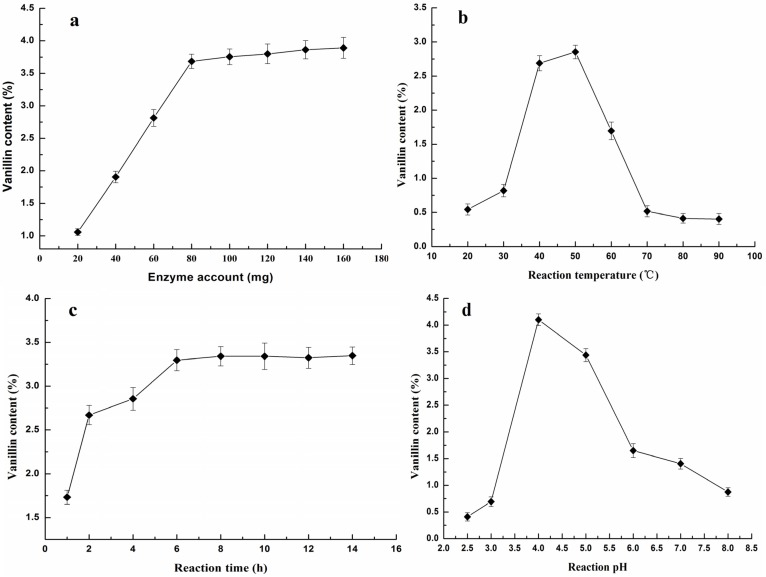
Effects of different (**a**) enzyme amounts, (**b**) reaction temperatures, (**c**) reaction times and (**d**) reaction pHs on vanillin content.

The added enzyme amount is an important factor that could remarkably influence the extraction efficiency [1,22]. The effect of pectinase amount on the vanillin yield is shown in Figure 1a. Different enzyme amounts were added at 20, 40, 60, 80, 100, 120, 140, 160 mg corresponding to 0.1%, 0.2%, 0.3%, 0.4%, 0.5%, 0.6%, 0.7%, 0.8% (w/w), respectively, when other variables were fixed at the extraction temperature of 50 °C, extraction time of 7 h, and extraction pH of 5. Pectinase consists of hemicellulase, cellulase and dextranase and can be used to degrade the pectin between the cells to make the enzymatic reaction between the glucovanallin substrate and β-glucosidase happen, which results in vanillin liberation. This effect doesn’t increase significantly as the pectinase amount increased until the pectinase amount reached 0.4%–0.6%, and the vanillin content reached its highest level of about 3.7% ± 0.05% when the enzyme amount was 80–120 mg. When more enzyme was added, the vanillin yield did not increase significantly (Figure 1a), indicating that 0.4% enzyme (80 mg) was sufficient to obtain a good vanillin content. All pectin substances were probably degraded so that most of glucovanallin substrate was hydrolyzed. The remaining glucovanallin substrate being not hydrolyzed may be ascribed to the effect of cellulose compartmentation or wrapping just as Perera and Owen indicated that the vanillin content could be increased after adding cellulase. Thus, it is suggested that an added enzyme amount in a range of 80–120 mg (0.4%–0.6%) was favorable for producing the vanillin from vanilla beans.

#### 2.1.2. Effect of the Temperature on the Vanillin Content

To investigate the effect of reaction temperature on vanillin extraction, the hydrolysis process was carried out at different temperatures of 20, 30, 40, 50, 60, 70, 80 and 90 °C while other variables (*i.e*., the enzyme amount, reaction time and hydrolyzed pH) were fixed at 7 h, 80 mg, and 5.0, respectively. As shown in Figure 1b, the vanillin content continues to increase as the extraction temperature increases from 20 to 50 °C, but rapidly decreases when the temperature continues to increase from 50 to 90 °C. The maximum vanillin content (2.85%) was obtained when the enzymatic temperature reached 50 °C. The increased reaction temperature from low temperatures resulted in faster and easier mass transfer of water-soluble components from the cell wall into the liquid, thus improving the extraction efficiency [20,24]. The reason for the decreased vanillin yield when the temperature increased from 50 to 90 °C was ascribed to the fact that the range from 50 °C to 90 °C is not the optimal temperature for the pectinase, whose optimal temperature is between 45–55 °C.

#### 2.1.3. Effect of the Time on the Vanillin Content

The effect of different times on vanillin production is shown in Figure 1c. Enzymatic hydrolysis was carried out for different time periods (1, 2, 4, 6, 8, 10, 12, 14, 16 h) while other extraction variables were fixed as follows: enzyme amount of 80 mg, reaction temperature of 50 °C, extraction pH of 5. The result showed that the vanillin content increased as the reaction time increased from 1 to 6 h, and the maximum vanillin content (3.34%) was obtained when the reaction time reached 8 h. After that the vanillin content remained at a steady value with the increasing hydrolysis time, and there was no obvious increase of the vanillin yield when the hydrolysis time exceeded 8 h (Figure 1c). This indicated that reaction time of 8 h was sufficient to extract the vanillin. Thus, extraction of 6–8 h was favorable for the production of vanillin in this experiment.

#### 2.1.4. Effect of the pH on the Vanillin Content

The vanillin content obtained after the enzyme hydrolysis at different pH values is shown in Figure 1d. The enzymatic process was carried out at pH 2.5, 3.0, 4.0, 5.0, 6.0, 7.0, and 8.0, while the other reaction parameters were kept at an enzyme amount of 80 mg, temperature of 50 °C, and time of 7 h, respectively. When the pH increased from 2.5 to 4.0, the vanillin content increased significantly from 0.41% to 4.1%, and thereafter it decreased to 0.87% as the pH increased from 4.0 to 8.0. The pH value can remarkably affect enzymatic activities due to the change of the enzyme’s spatial structure and conformation [22]. Thus, the appropriate pH of the pectinase for this extraction was determined in the range of 3.0–5.0.

### 2.2. Optimization of Enzymatic Conditions for Vanilla Extraction Using RSM

#### 2.2.1. Statistical Analysis and the Model Fitting

Response surface methodology (RSM) is a method that can more efficiently collect statistical and mathematical parameters for developing, improving, and optimizing processes. It is more suitable to extract multivariate data which is obtained from properly designed experiments to simultaneously determine multivariable equations [18,19,21,23].

Table 2 lists the design matrix and the corresponding results of CCD experiment which was consisted of four factors, five levels, and six center point replicates. The results showed that the vanillin content varied from 1.19% to 4.53%. The predicted vanillin production values based on the model are also shown in Table 2, which indicates that there is a close agreement between the experimental and predicted values. The F-test and *p* values were used to identify the effect of each factor on the enzymatic hydrolysis for vanillin extraction, and the analysis of variance (ANOVA) for the response surface quadratic model is shown in Table 3. The results showed that the model was adequate for model prediction within the range of the experimental variables because the determination coefficient was *R^2^* = 0.9973, only 0.27% of the total variations were not explained by the model. The value of the adjusted determination coefficient (Adj. *R^2^* = 0.9949) also confirmed that the model was highly significant. CV is a measure expressing the standard deviation as a percentage of the mean, smaller value of CV gives better reproducibility [20]. If a CV is higher than 10, it indicates that variation in the mean value is high and does not satisfactorily develop an adequate response model [25]. In this research, a very low value of coefficient of the variation (CV = 1.72, Table 3) clearly indicated a very high degree of precision and a good reliability for the experimental values. In addition, the low PRESS value (0.21) suggests for an adequacy of the fitted quadratic models for the predictive applications (Table 3). Adequate precision measures the signal-to-noise ratio, in which a ratio greater than 4 is desirable and adequate for model discrimination [19]. In this research, the signal-to-noise ratio value of 76.867 represented a very good signal-to-noise ratio.

**Table 2:** Response surface central composite design (uncoded) and results for vanillin content (%).

Run	X_1_ (mg)	X_2_ (°C)	X_3_ (h)	X_4_ (pH)	Vanillin (%)	
					Predicted	Actual
1	80.0	50.0	7.0	5.0	3.26	3.31
2	90.0	47.5	6.5	4.5	3.81	3.85
3	60.0	50.0	7.0	4.0	2.70	2.67
4	70.0	52.5	6.5	4.5	3.49	3.48
5	90.0	52.5	6.5	3.5	2.57	2.59
6	70.0	47.5	6.5	3.5	2.43	2.48
7	80.0	45.0	7.0	4.0	3.67	3.68
8	70.0	47.5	7.5	4.5	3.51	3.48
9	90.0	52.5	7.5	3.5	3.10	3.14
10	80.0	50.0	7.0	4.0	4.45	4.49
11	80.0	50.0	7.0	4.0	4.45	4.39
12	80.0	50.0	7.0	4.0	4.45	4.49
13	70.0	52.5	6.5	3.5	1.94	1.98
14	70.0	47.5	7.5	3.5	2.77	2.78
15	80.0	50.0	7.0	4.0	4.45	4.53
16	80.0	50.0	7.0	4.0	4.45	4.38
17	80.0	50.0	8.0	4.0	3.99	4.02
18	90.0	47.5	7.5	4.5	3.96	3.92
19	70.0	52.5	7.5	3.5	2.17	2.12
20	90.0	47.5	6.5	3.5	2.85	2.81
21	80.0	55.0	7.0	4.0	3.10	3.08
22	90.0	52.5	6.5	4.5	3.84	3.84
23	80.0	50.0	6.0	4.0	3.61	3.56
24	100.0	50.0	7.0	4.0	3.77	3.79
25	70.0	52.5	7.5	4.5	3.22	3.27
26	90.0	52.5	7.5	4.5	3.88	3.83
27	90.0	47.5	7.5	3.5	3.49	3.49
28	80.0	50.0	7.0	3.0	1.24	1.19
29	70.0	47.5	6.5	4.5	3.67	3.65
30	80.0	50.0	7.0	4.0	4.45	4.41

**Table 3:** Analysis of variance for the fitted models.

	Source	Degree of freedom	Coefficient	Sum of square	Mean square	F-Value	*p*-Value
Vanillin content (%)	Model	14		19.55	1.40	401.75	<0.0001
	Residual	13		0.052	0.0035		
	Lack of fit	10		0.031	0.0031	0.75	0.6767 ns
	Pure error	3		0.021	0.0042		
	Total	29		19.60			
	R^2^		0.9973				
	Adj-R^2^		0.9949				
	CV		1.72				
	PRESS		0.21				
	Standard deviation		0.059				
	Adequate precision		76.867				

If the lack of fit is significant, the model will fail to represent the data in the experimental domain at which points were not included in the regression [22]. The ANOVA showed that both the F-value of 0.75 and *p*-value of 0.6767 for the vanillin extraction implied that the lack of fit of the model was not significant to the pure error due to noise at 95% confidence level, which means that the model has represented the data satisfactorily. All these statistical parameters demonstrated the reliability of the model.

The *p* values were used as a tool to check the significance of each of the coefficients which, in turn, are necessary to understand a pattern of mutual interactions between the test variables. Smaller the magnitude of *p* value, more significant is the corresponding coefficient [23]. The significance of each coefficient of Equation. (2) was determined using *p*-value and presented in Table 4, where linear coefficients (X_1_, X_2_, X_3_, X_4_), quadratic term coefficient (X_12_, X_22_, X_32_, X_42_) and cross product coefficients (X_1_X_2_, X_1_X_3_, X_1_X_4_, X_2_X_4_, X_3_X_4_) were significant with very small *p* values (*p* < 0.01). In contrast, the other term coefficients were not significant (*p* > 0.05).

**Table 4:** Estimated regression model of relationship between response variables (vanillin %) and independent variables (X_1_, X_2_, X_3_, X_4_).

Variables	DF	SS	MS	F-value	*p*-value
X_1_	1	1.74	1.74	499.55	<0.0001
X_2_	1	0.49	0.49	139.86	<0.0001
X_3_	1	0.22	0.22	62.09	<0.0001
X_4_	1	6.12	6.12	1,760.23	<0.0001
X_1_X_1_	1	2.51	2.51	723.34	<0.0001
X_1_X_2_	1	0.046	0.046	13.33	0.0024
X_1_X_3_	1	0.092	0.092	26.53	0.0001
X_1_X_4_	1	0.075	0.075	21.63	0.0003
X_2_X_2_	1	1.93	1.93	554.04	<0.0001
X_2_X_3_	1	0.011	0.011	3.13	0.0970
X_2_X_4_	1	0.097	0.097	27.91	<0.0001
X_3_X_3_	1	0.73	0.73	210.00	<0.0001
X_3_X_4_	1	0.25	0.25	70.83	<0.0001
X_4_X_4_	1	8.26	8.26	2,375.51	<0.0001

#### 2.2.2. Analysis of Response Surfaces

**Figure 2:**
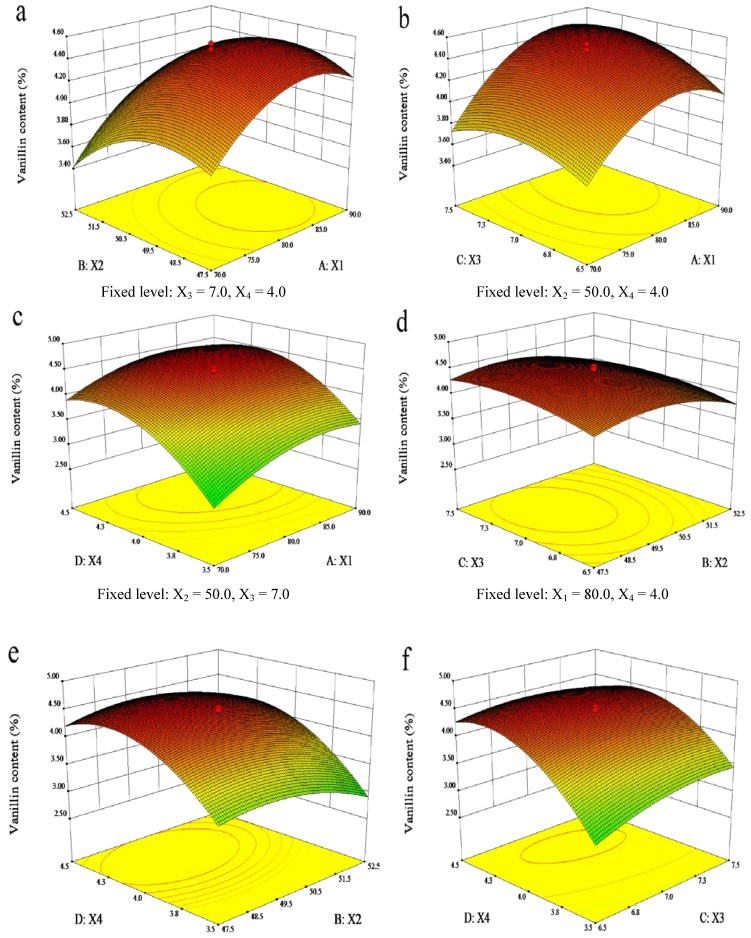
Response surface (3-D) showing the effects of the (X_1_) enzyme amount, (X_2_) reaction temperatures, (X_3_) reaction time, and (X_4_) reaction pH on the response (Y) vanillin content.

**Figure 3:**
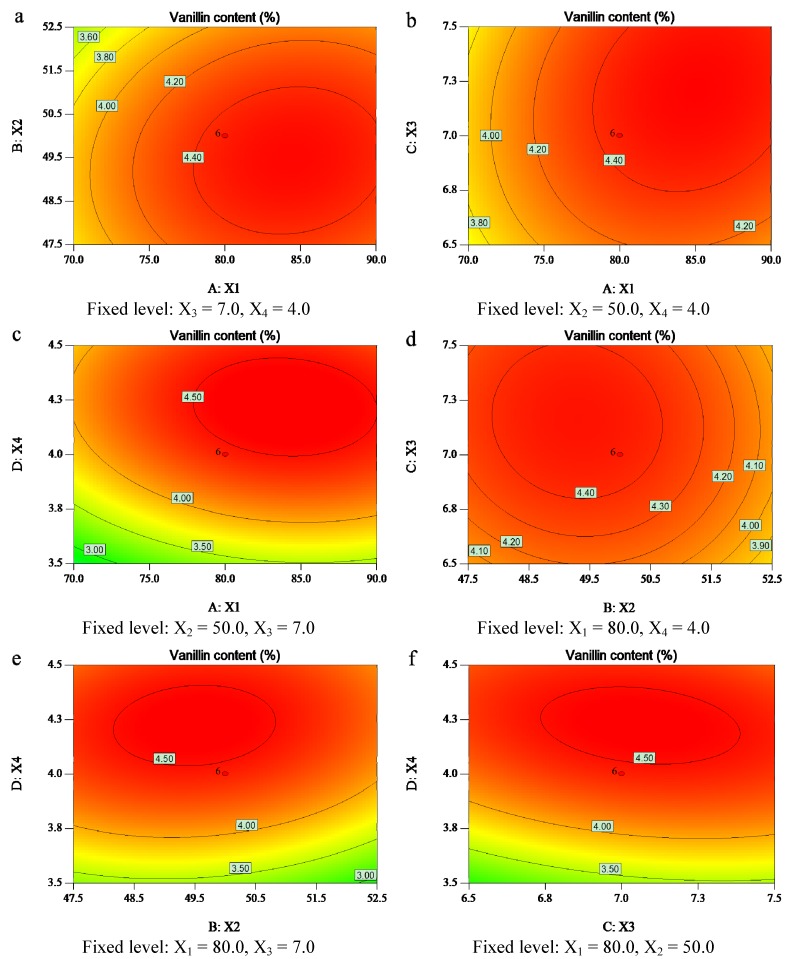
Contour plots showing the effect of the effects of the (X_1_) enzyme amount, (X_2_) reaction temperature, (X_3_) reaction time, and (X_4_) reaction pH on the response (Y) vanillin content.

In order to determine the optimal levels of each variable for a maximum vanillin production, response surface and contour plots were constructed by the Design-Expert software to plot the response (vanillin content) against any two independent variables. In the response surface plot and contour plot, the data were generated through keeping two variables at their respective zero level (central value of the testing ranges) while changing the other two variables within the experimental range. The graphical representation was used to accomplish a better understanding of the interactions between the variables. Response surface methodology plays a key role in identifying the optimum values of the independent variables efficiently, under which dependent variables could arrive the maximum response. The results of vanillin content affected by enzyme amount, reaction temperature, time, and pH are presented in Figure 2 and Figure 3.

Figure 2a and Figure 3a are profiled the vanillin content as a function of the added enzyme amount and reaction temperature at the fixed hydrolysis time (7.0 h) and pH (4.0). A quadratic effect was observed for both variables, although enzyme amount had greater influence on the response. This indicated that the vanillin content increased with the increase of enzyme amount from 70.0 to 84.2 mg when the vanillin content reached its maximum plateau region. In contrast, the content increased with the increase of temperature from 45.0 to 49.6 °C while it decreased with the continued increase of the reaction temperature from 49.6 to 55.0 °C. This phenomenon was ascribed to the fact that higher temperatures reduced the enzyme activity.

The vanillin yield could also be affected by different reaction time and enzyme amount (Figure 2b and Figure 3b), when the other two variables (reaction temperature and pH) were fixed at 50 °C and 4.0, respectively. The vanillin content was found to increase rapidly with increase of enzyme amount from 70.0 to 84.2 mg, as it did for the hydrolysis time increase from 6.0 to 7.1 h. Then, vanillin content did not increase further. It can be seen that the maximum vanillin content can be achieved when the reaction time and enzyme amount are around 7.1 h and 84.2 mg, respectively.

Figure 2c and Figure 3c show the 3D response surface plot and the contour plot at different reaction pH values and enzyme amounts at fixed reaction temperature and time. It was found that the hydrolysis pH had a positive synergistic effect with the enzyme amount. Likewise, the vanillin content was found to increase rapidly with the increase of reaction pH from 3.0 to 4.2, and then decrease rapidly from 4.2 to 5.0. It indicated that the maximum vanillin content could be achieved when the reaction pH and enzyme amount were at the 4.2 and 84.2 mg levels, respectively.

Figure 2d and Figure 3d were the 3-D response surface plot and the contour plot that were developed for the vanillin content with different reaction temperatures and times at a fixed enzyme amount of 80.0 mg and pH 4.0. It was observed that the maximum vanillin content could be achieved when reaction temperature and time were at the threshold levels of 49.5 °C and 4.2 h, respectively.

The 3D response surface plot and the contour plot based on the independent variable reaction pH and temperature are shown in Figure 2e and Figure 3e, while the other two independent variables, enzyme amount and reaction time were kept at 80 mg and 7.0 h, respectively. It was found that the effect of pH on the vanillin content was more pronounced at lower temperatures, and the maximum vanillin content can be achieved when reaction pH and temperature were kept at the threshold level of around 4.2 and 49.5 °C, respectively.

Figure 2f and Figure 3f present the profiles of the 3D response surface plot and the contour plot at different reaction time and pH when the enzyme amount (84.2 mg) and temperature (50 °C) were fixed. The content increased with the reaction time from 6.5 to 7.1 h, and reached the maximal plateau region when the reaction pH was at the critical level of 4.2.

It can be concluded from the analysis of Figure 2 and Figure 3 that the optimal conditions for vanillin production from vanilla beans are an enzyme amount of 84.2 mg, reaction temperature of 49.5 °C, time of 7.1 h, and pH of 4.2. It was found that among the four extraction parameters enzyme amount was the most significant factor that affected the vanillin content, followed by reaction pH, temperature and time based on the regression coefficients significance of the quadratic polynomial model (Table 3) and gradient of slope in the 3-D response surface plots (Figure 2).

### 2.3. Analysis of the Model Adequacy

**Figure 4:**
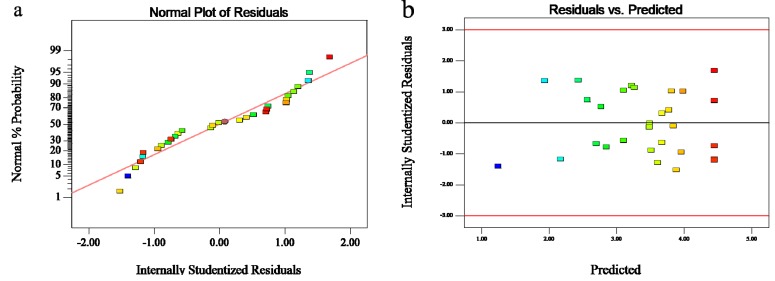
(**a**) Normal probability of internally studentized residuals. (**b**) Predicted values *versus* experimental values of vanillin content (%).

If a model does not show an adequate fit, it will lead to poor or misleading analysis and optimization results. Therefore, it is necessary to check the model adequacy in a real system. As indicated by one published report [25], model adequacy could be judged by the residuals from the least squares fit. In this study, Figure 4a shows the normality assumption by constructing a normal probability plot of the residuals, indicating that the normality assumption was satisfied when the residual plot approximated along a straight line. Figure 4b shows the plot of residuals *versus* the predicted response.

As shown in Figure 4b, the residuals are distributed randomly on the plot, suggesting that the variance of the original observation is constant for all values of Y [18]. From the plots of Figure 4a,b, it was concluded that the model in this study is adequate to describe the vanillin content determined by the response surface.

### 2.4. Verification of Predictive Model

To confirm the validity of the suggested mathematical model, an additional experiment was conducted using the predicted optimal conditions. The optimal conditions and predicted optimum response values are listed in Table 5. The optimal condition was an enzyme amount of 84.15 mg, enzymatic temperature of 49.55 °C, time of 7.13 h and pH of 4.20. Under these conditions, the theoretical vanillin production was 4.63% according to the RSM. However, considering the actual production, the optimal conditions can be modified to an enzyme amount of 84 mg (0.4%), reaction temperature of 50 °C, time of 7.1 h and pH of 4.2, respectively. The calculated value of 4.63% was not significantly different from the mean value (4.62%) of three experimental tests, suggesting that the RSM model was adequate for the expected optimization (Table 5). This result of analysis indicated that the experimental values were in good agreement with the predicted ones, and also demonstrated the validation of the RSM model and indicated that the model was adequate for the extraction process (Table 5).

**Table 5:** Predicted and experimental values of the responses at optimum conditions.

	X_1_ (mg)	X_2_ (°C)	X_3_ (h)	X_4_ (pH)	Vanillin (%)
Predicted	84.15	49.55	7.13	4.20	4.63
Experimental ^a^	84	50	7.1	4.2	4.62±0.14

^a^ Mean ± standard deviation (n = 3); X_1_, pectinase amount; X_2_ temperature; X_3_ time; X_4_ pH.

### 2.5. Effects of Different Treatments

As shown in Table 1, the major constituents of vanilla extract from different treatments were *p*-hydroxybenzoic acid, *p*-hydroxybenzaldehyde, vanillic acid, and vanillin. Besides, glucovanillin was detected in the four samples. This indicated that there were highly significant (*p* < 0.05) differences among the three treatments as well as constituents in the vanilla extracts by t test (Table 1). Compared to the FTV, the three vanilla extract showed higher levels of flavor constituents. Among them, the content of single most characteristic component of vanilla flavor—vanillin—had its highest level in the PVE (4.62%). Compared to the traditional curing process of SVE (43.58%) and viscozyme extraction of VVE (59.31%), the rate of transformation of PVE (87.79%) increased by 43.21% and 28.48%, and as a result, the vanillin production significantly increased the yield by 133.85% and 96%, respectively.

Enzymatic pretreatment could facilitate the vanillin extraction [3]. In addition, tissue disruption by freezing and thawing together with added hydrolyzing enzymes liberated a higher level of vanillin from vanilla beans [4,5], which was also confirmed by our study that a highest rate of transformation from glucovanillin to vanillin was achieved when the enzyme amount, temperature, time, and pH were controlled at 84.2 mg, 49.5 °C, 7.1 h, and 4.2.

In this work glycosidase wasn’t added based on the following two considerations: first, the level of internal β-glucosidase in the green vanilla pods was enough for glucovanillin transformation as shown by Ansaldi *et al.* and Wang *et al.* [13,26]. Second, the β-glucosidase level only has an effect on glucovanillin hydrolysis kinetics but is not the limiting factor for complete hydrolysis just as Odoux *et al.* indicated [12]. External glycosidase may show some positive effects in the extraction process of vanillin from natural green vanilla beans such as increasing the rate of glucovanillin hydrolysis and shorting the extraction time. The productivity of vanillin and the cost of different enzyme used in extraction process will be further investigated by adding glycosidase.

As one trend to b examined in future work, enzyme immobilization technology could be one way to further improve the process of pectinase-assisted extraction combined with pre-freezing and thawing. We will thus further study the effect of enzyme immobilization technology on the properties of the pectinase based on product cost in details.

## 3. Experimental

### 3.1. Materials

Mature green vanilla beans (*Vanilla planifolia* Andrews) and its cured vanilla were harvested and obtained from the Spice and Beverage Research Institute (Hainan, China). Only the mature vanilla beans with a slight yellow at the blossom end and without any splitting of the ends were used in subsequent experiments. The water content of the green and cured beans was determined to be 87.24% and 14.93%, respectively, according to a published method [5]. Uronic acid and galacturonan contents were 1.91% and 214 mg of per gram cell wall (dry basis, db), respectively, determined by a published method [10]. Pectinase (30 U/mg) and viscozyme (100 FBG/g) were purchased from Aladdin Agent Co., Ltd (Shanghai, China) and Novo Nordisk (Copenhagen, Denmark), respectively. The pectinase consisting of hemicellulase, cellulase and dextranase from *Aspergillus niger* can be used to degrade pectin between the cells to make the enzymatic reaction between the glucovanallin substrate and β-glucosidase happen. The purity of pectinase was above 96%. Vanilla standards (glucovanillin, vanillin, vanillic acid, *p*-hydroxybenzaldehyde, *p*-hydroxybenzoic acid) were purchased from Sigma Chemical Company (St. Louis, MO, USA). Methanol (HPLC grade) and other chemicals (analytical grade) were purchased from Shanghai Chemical Reagent Co., Ltd. (Shanghai, China).

### 3.2. Freezing and Thawing

The operation was conducted according to a published report [4] with some modifications. Fresh vanilla beans (FVB) were vacuum packed in bags (five to a bag), frozen at −40 °C for 24 h, and then thawed at 37 °C in a covered water bath, which was named frozen-thawed vanilla (FTV).

### 3.3. Soxhlet Extraction

Soxhlet extraction was operated according to the reported method [5] with some modifications as follows: powdered cured vanilla beans (3 g) were weighed in a cellulose thimble and placed in a Soxhlet apparatus. The vanilla compounds were extracted using 47.5% *v/v* aqueous ethanol solution at 95 °C for 7.1 h. The vanilla extract from this method was called the Soxhelt-vanilla extract (SVE). The amounts of major constituents in vanilla extracts were quantified by HPLC after the extraction.

### 3.4. Enzyme-Assisted Extraction

Vanilla beans were chopped in pieces of 0.5 cm in length and placed in a laboratory grinder where distilled water (1:1, *w/w*) was added. Then they were separated into equal portions (20 g each). For the first portion, 1% *v/v* of viscozyme was added and incubated at 50 °C for 7.1 h. Other pretreated samples (20 g) including the control sample (FTV) were extracted at a designated enzyme concentration, reaction temperature, time, and pH. All of the reaction contents were kept in suspension by agitation using a magnetic stirrer. The extraction ended 30 min later after the addition of ethyl ethanol to reach 47.5% v/v concentration in the enzyme reaction mixture. The vanilla extracts obtained from the uses of viscozyme and pectinase were called VVE and PVE, respectively. The amounts of major constituents in the vanilla extracts were quantified by HPLC after the viscozyme and pectinase hydrolysis and extraction.

### 3.5. Experimental Design and Data Analysis

After determining the preliminary range of extraction variables through a single-factor test, the effects of four variables (pectiase amount, reaction temperature, time and pH) on the vanillin production were studied through the central composite design (CCD) and response surface methodology (RSM). These four independent variables were investigated at five different coded levels (*i.e*., −2, −1, 0, +1 and +2) selected on the basis of single-factor test, which suggested the optimal ranges that are presented in Table 6.

**Table 6:** Independent variables and their levels used in the response surface design.

Independent variables	Factor level				
Coded levels	−2	−1	0	1	2
X_1_ (mg)	60	70	80	90	100
X_2_ (°C)	45.0	47.5	50.0	52.5	55.0
X_3_ (h)	6.0	6.5	7.0	7.5	8.0
X_4_	3.0	3.5	4.0	4.5	5.0

X_1_, pectinase amount; X_2_ temperature; X_3_ time; X_4_ pH.

For statistical calculation, the variables were coded according to equation (1):

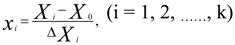
(1)
where *x_i_* is the coded independent factor, *X_i_* is the actual value of the independent variable, *X_0_* is the actual value of *X_i_* at the center point and *ΔX_i_* is the step change value.

Thirty experimental points (*i.e.*, 16 factorial points, eight axial points, and six center points) were carried out according to the parameters listed in Table 6. Triplicates were performed at all design points in a randomized order. The response values in the Table 6 are the averages of triplicates.

A second-order polynomial regression model was used to express the vanillin content as a function of the independent variables as follows:

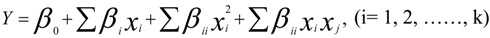
(2)
where Y represents the response variable, β_0_ is the constant, β_i_, β_ii_ and β_ij_ are the coefficients for the linear, quadratic and interaction effects, respectively. x_i_ and x_j_ are the levels of the independent variables.

The second-order polynomial model given by the Equation (2) was fitted to the experimental data to obtain the regression equations. Analysis of the experimental design and calculation of predicted data were performed using the Design Expert software (Version 8.05 b, Stat-Ease Inc., Minneapolis, MN, USA). The significant effect was separated according to analysis of variance (ANOVA) as non-significant (*p* > 0.05) or significant (*p* < 0.05). The fitted polynomial equation is further profiled in 3D surface and contour plots in order to visualize the relationship between the response and experimental levels of each variable and to deduce the optimal conditions [17,21]. Subsequently, three additional experiments were conducted to verify the validity of the statistical experimental strategies.

### 3.6. Determination of the Major Constituents in Different Treatment Samples

The chemical quantification was determined on the base of dry weight basis according to previously described methods [1,2] with a slight modification. The chemicals were determined by a HPLC system (Agilent 1260 series HPLC, Agilent Technologies, Palo Alto, CA, USA), which was equipped with a Zorbax Eclipse Plus C18 (reverse Phase, 4.6 mm × 100 mm, 3.5 μm) analytical column (Agilent Technologies) maintained at 30 °C. Isocratic elution condition using the solvent ratio of acetonitrile to water (10:90 *v/v)* was carried out at a flow rate of 1 mL/min. The standard solutions (20, 40, 60, 80, 100 and 120 μg/mL) were used to prepare the standard curves and analyzed in triplicates by injecting 5 μL of the sample into the HPLC system. A UV detector at 280 nm wavelength was used for the chemical detection. The samples were filtered through 0.45 μm membrane filters prior to the HPLC analysis. The collected data were subjected to calculation of the mean value and the calibration curve was created by plotting the concentrations of the injected amount against the corresponding peak areas.

### 3.7. Statistical Analysis

The mean values, standard deviations and significant differences of the data were calculated and reported using SPSS 12.0.1 (SPSS Inc., Chicago, IL, USA). Whenever reported differences were significant, a confidence level of 95% was considered. The data reported in all of the tables were the average of triplicate observations.

## 4. Conclusions

RSM was proven to be useful for optimizing vanillin production from vanilla beans. The coefficients of determination (0.9973) and the probability value (*p* < 0.0001) for the regression model in this study demonstrated a very high credibility for predicting the responses. The enzymatic hydrolysis after the freezing treatment showed significant effects on the vanillin content. The optimum set of independent variables was graphically profiled to determine the desirable level of vanillin production. The maximal value of the vanillin production (4.62% ± 0.14%) was obtained when the enzyme amount, enzymatic temperature, hydrolysis time, and pH were controlled at 84 mg, 50 °C, 7.1 h, 4.2, respectively, which was validated at the optimal condition with a vanillin production at 4.63%. The present study indicated that the optimized pectinase-assisted extraction combined with pre-freezing and thawing can be used for the improved transformation of glucovanillin to vanillin and the production of natural vanillin from green vanilla.
